# Mechanisms of low susceptibility to the disinfectant benzalkonium chloride in a multidrug-resistant environmental isolate of *Aeromonas hydrophila*

**DOI:** 10.3389/fmicb.2023.1180128

**Published:** 2023-06-02

**Authors:** Luz Chacón, Benno Kuropka, Enrique González-Tortuero, Frank Schreiber, Keilor Rojas-Jiménez, Alexandro Rodríguez-Rojas

**Affiliations:** ^1^Evolutionary Biology, Institut für Biologie, Freie Universität Berlin, Berlin, Germany; ^2^Health Research Institute, University of Costa Rica, San José, Costa Rica; ^3^Division of Biodeterioration and Reference Organisms (4.1), Department of Materials and the Environment, Federal Institute for Materials Research and Testing (BAM), Berlin, Germany; ^4^Institute of Chemistry and Biochemistry, Freie Universität Berlin, Berlin, Germany; ^5^School of Science, Engineering, and Environment (SEE), University of Salford, Manchester, United Kingdom; ^6^Escuela de Biología, University of Costa Rica, San José, Costa Rica; ^7^Small Animal Internal Medicine, Clinic for Small Animals, University of Veterinary Medicine (Vetmeduni), Vienna, Austria

**Keywords:** benzalkonium chloride, wastewater, biocide, quaternary ammonium disinfectants, low-susceptibility, primary response

## Abstract

Excessive discharge of quaternary ammonium disinfectants such as benzalkonium chloride (BAC) into aquatic systems can trigger several physiological responses in environmental microorganisms. In this study, we isolated a less-susceptible strain of *Aeromonas hydrophila* to BAC, designated as INISA09, from a wastewater treatment plant in Costa Rica. We characterized its phenotypic response upon exposure to three different concentrations of BAC and characterized mechanisms related to its resistance using genomic and proteomic approaches. The genome of the strain, mapped against 52 different sequenced *A. hydrophila* strains, consists of approximately 4.6 Mb with 4,273 genes. We found a massive genome rearrangement and thousands of missense mutations compared to the reference strain *A. hydrophila* ATCC 7966. We identified 15,762 missense mutations mainly associated with transport, antimicrobial resistance, and outer membrane proteins. In addition, a quantitative proteomic analysis revealed a significant upregulation of several efflux pumps and the downregulation of porins when the strain was exposed to three BAC concentrations. Other genes related to membrane fatty acid metabolism and redox metabolic reactions also showed an altered expression. Our findings indicate that the response of *A. hydrophila* INISA09 to BAC primarily occurs at the envelop level, which is the primary target of BAC. Our study elucidates the mechanisms of antimicrobial susceptibility in aquatic environments against a widely used disinfectant and will help better understand how bacteria can adapt to biocide pollution. To our knowledge, this is the first study addressing the resistance to BAC in an environmental *A. hydrophila* isolate. We propose that this bacterial species could also serve as a new model to study antimicrobial pollution in aquatic environments.

## 1. Introduction

Antimicrobial agents used for disinfection in domestic, industrial, and medical scenarios are deposited in wastewater and can be released into the environment after treatment (Tezel and Pavlostathis, 2015). One of the most used disinfectants is the quaternary ammonium compound benzalkonium chloride (BAC), a cationic surfactant with biocidal properties against viruses, bacteria, and some fungi, including yeast. BAC has homologs with varying alkyl chain lengths and is used in large quantities, with over 450,000 kg manufactured or imported to the United States annually. As a result, the American Environmental Protection Agency (EPA) has placed BAC on its High Production Volume List (Barber and Hartmann, 2022). During the COVID-19 pandemic, the consumption of quaternary ammonium compound (QAC) products containing BAC increased significantly due to their widespread use. Remarkably, in the last few years, the mass load of QAC compounds in wastewater increased by 331% compared to pre-pandemic levels (Mohapatra et al., 2023).

BAC can remain in biosolids, such as activated sludge, for extended periods because a significant proportion of it gets integrated into the biomass, with nearly 95% of the compound adsorbed in just a few hours (Zhang et al., 2011). Furthermore, BAC degradation in solids is approximately 20 times slower than in the liquid phase, exacerbating the situation (Zhang et al., 2011; Barber and Hartmann, 2022). Previous studies have demonstrated that BAC is present in digested and sewage sludges used for fertilization purposes as land biosolids (Östman et al., 2017; U.S. Environmental Protection Agency, 2018). The biocidal effect of BAC begins when it interacts with cell membranes, leading to perturbation of the lipid bilayer and a progressive leakage of cytoplasmic materials outside the cell. BAC can also bind to anionic sites on surface membranes and induce osmoregulatory losses. BAC affects bacteria's respiration, solute transport, and cell wall biosynthesis at high concentrations. One common mechanism of microbial death is the solubilization of the cell membrane, which leads to the release of cellular contents into the extracellular space (Gilbert and Moore, 2005). Although QACs are commonly associated with damaging cell membranes, recent research suggests that their effects may extend beyond the surface to intracellular targets (Knauf et al., 2018). To survive under BAC exposure, some intrinsic responses have been described in Gram-negative bacteria, including structural cell membrane modification, increased activity of efflux pumps to extrude biocides, biofilm formation, and formation of persister cells. Some of these adaptations can be related to cross-tolerance to other types of QAC and antibiotics (Mohapatra et al., 2023).

The most common bacterial models used to study the development of resistance to BAC are reference strains of *Pseudomonas aeruginosa* (Loughlin et al., 2002; Edirsana et al., 2014; El-Banna et al., 2019), isolates from food of *Listeria monocytogenes* (Romanova et al., 2006; Conficoni et al., 2016; Yu et al., 2018), and other clinically relevant isolates (Mahzounieh et al., 2014; Worthing et al., 2018; Abdelaziz et al., 2019). However, it is recognized that antimicrobial resistance originates in the environment, where waters, soil, and other sites can provide an unmatched gene pool with a great diversity that can exceed that of the animal and human microbiota. Consequently, antimicrobial pollution contributes to the mutation-based evolution of resistance (Larsson and Flach, 2022). For this reason, it is necessary to address the resistance profile and response to antibiotics and biocides to understand their impact on environmentally-relevant bacterial species. Hypothesis-independent OMIC approaches allow the inference of the molecular mechanisms underlying antimicrobial resistance in such environmental bacteria without building these strains into elaborate model systems.

During a microbiological sampling campaign at a wastewater treatment plant near San José, Costa Rica, we isolated and identified a bacterial environmental isolate as *A. hydrophila* through sequencing. We designated this strain as *A. hydrophila* INISA09. This bacterial species belongs to the Phylum Proteobacteria, Class Gammaproteobacteria, Order Aeromonadales, and Family Aeromonadaceae. It is a Gram-negative bacillus positive for oxidase and catalase, a glucose fermenter, and it can reduce nitrates to nitrites. Different strains of this species are ubiquitous and typically presents in aquatic environments (Fernández-Bravo and Figueras, 2020). *A. hydrophila*'s ability to colonize different natural and urban aquatic ecosystems and its capacity as an opportunistic human and animal pathogen makes it a desirable candidate for evaluating the dissemination of antimicrobial resistance (Igbinosa and Okoh, 2012; Karkman et al., 2018).

This study aims to characterize a naturally occurring bacterial isolate, a strain of *A. hydrophila* named INISA09, from a tropical activated sludge and to understand its response to increasing benzalkonium chloride (BAC) concentrations using a quantitative label-free proteomics approach.

## 2. Materials and methods

### 2.1. Bacteria and growth conditions

An *A. hydrophila* INISA09 strain, isolated from activated sludge in Costa Rica, was used as a bacterial model for all experiments with BAC. The isolate was recovered after an activated sludge exposure to BAC (Chacón et al., 2021) in trypticase soy agar (TSA) (Oxoid^®^, United States) enriched to 25 mg/L BAC (≥95.0% Fluka 12060 Sigma, United States). The *16S rRNA* gene was sequenced to preliminarily identify the bacterial species. LB Lennox medium (Carl Roth, Germany) was used for all experiments. The growing temperature in all experiments was 30°C, and when shaking conditions were required, the shaker incubator was set to 200 rpm.

### 2.2. Genome sequencing

DNA extraction was conducted with DNeasy Blood & Tissue Kit (Qiagen, USA) using the pre-treatment step for Gram-negative bacteria recommended by the manufacturer. The Illumina NovaSeq platform at the company Novogene (Hong Kong) was used for DNA sequencing. Sequence quality was examined using FastQC (Brown et al., 2017). The removal of low-quality reads and adapters from the paired ends for all sequences was filtered by Trim Galore v. 0.6.6 (Krueger, 2020) using a Phred quality cutoff of 30. Then, human reads were discarded by mapping them against the Genome Reference Consortium Human Build 38 (GRCh38; Schneider et al., 2017) using Bowtie2 v. 2.4.2 (Langmead and Salzberg, 2012) with default parameters. The reads were mapped against 52 different sequenced *A. hydrophila* strains (Supplementary Table S1) using Bowtie2 with default parameters which were used to remove all potential bacterial contamination. After that, for the *de novo* assembly, Shovill v. 1.1.0 (Seemann, 2020) was executed using SPAdes v. 3.15.0 (Bankevich et al., 2012) as the main assembler and with default parameters. The resulting contigs were scaffolded against *A. hydrophila* ATCC 7966, the recommended reference strain by the European Committee on Antimicrobial Susceptibility Testing (EUCAST), and the other strains detailed in Supplementary Table S1, using CSAR (Chen et al., 2018). Duplicated entries were removed using SeqKit v. 2.3.1 (Shen et al., 2016). All resulting scaffolds were annotated with Prokka v.1.14.6 (Seemann, 2014) using the bacterial genetic code for gene and tRNA prediction (--*gcode 11*), allowing the search of non-coding RNA elements (*--rfam*), predicting the Gram-negative signal peptides in the CDSs (*--gram neg*), and complying with the GenBank specifications for the file formatting (*--compliant*). Fast average nucleotide identity (ANI) (Jain et al., 2018) was used to detect the conservation degree among all *A. hydrophila* strains mentioned in this study, including *A. hydrophila* INISA09. ANI analysis was visualized using R v. 4.2.2. (R Development Core Team, 2015) with the libraries *ape* v. 5.7.1 (Paradis and Schliep, 2019) and *phangorn* v. 2.11.1 (Schliep, 2011; Schliep et al., 2017) altogether with FigTree v. 1.4.4 (http://tree.bio.ed.ac.uk/software/figtree) for the phylogram, and the library *ComplexHeatmap* v. 2.14.0 (Gu et al., 2016) for the heatmap. Snippy v. 4.6.0 (Seemann, 2015) was used to detect the conservation degree and genomic variation between *A. hydrophila* INISA09 and *A. hydrophila* ATCC 7966. Visualization of the genome was performed using the CGView server (Stothard and Wishart, 2005), with CRISPRCasFinder (Couvin et al., 2018) and CARD (Alcock et al., 2019) modules for the visualization of CRISPR-Cas associated elements and antibiotic resistance genes, respectively. This whole-genome shotgun project has been deposited at DDBJ/ENA/GenBank under the accession JAPKVL000000000. The version described in this study is JAPKVL010000000.1.

### 2.3. BAC minimum inhibitory concentration (MIC) and dose–Response curve determination

A bacterial inoculum of 10^6^ colony-forming units per milliliter (CFU/mL) was used to start the minimum inhibitory concentration assay (MIC). The MIC was determined with the broth microdilution method (Andrews, 2001) with slight modifications. A volume of 200 μL was used as the total volume per well in 96-well polypropylene microtiter plates (Greiner Bio-One, Germany), and the MIC was assessed after 36 h at 30°C of incubation in a microtiter plate reader (Biotek Epoch 2). The MIC was defined as the lowest concentration inhibiting liquid culture growth. The BAC (Fluka^®^ Analytical, Germany) concentrations tested were 0.7 μg/mL, 7.0 μg/mL, 14.0 μg/mL, 19.0 μg/mL, 23.0 μg/mL, 28.0 μg/mL, 33.0 μg/mL, 38.0 μg/mL, 76.0 μg/mL, and 380.0 μg/mL. For each concentration, seven replicates were tested. Additionally, as control, one well was used to blank for BAC + LB medium turbidity, seven wells were used as growing control (without BAC), and seven wells were used as LB medium turbidity control. Optical density measurements were collected in the plate reader using the software Gen5 3.09. A growth curve analysis was performed using the Growthcurver package for R (Sprouffske and Wagner, 2016). For analyzing differences in growing parameters, a Kruskal–Wallis test was conducted with software R version 4.1.1 (2021-08-10) (R Development Core Team, 2015). For the dose–response curve, a growth rate was calculated from OD_600_ measurement, which was taken every 10-min interval in LB medium (with and without BAC), using an algorithm described by Swain (Swain et al., 2016) and implemented in Python 3.6. Finally, the time–kill curve to BAC was determined by exposing exponential phase bacteria at a density of ~1x10^9^ CFU/mL to 40.0 μg/mL (data not shown).

### 2.4. Antibiotic susceptibility profile

Approximately 10^6^ CFU/mL were inoculated onto LB Lennox Agar plates (LB medium, Carl Roth, Germany; plus agar 1.5%), following the disk diffusion method previously described (CLSI, 2018). Filter disks were impregnated with 10 μL of the following antibiotics: ciprofloxacin (1 mg/mL), streptomycin (50 μg/mL), ceftazidime (50 μg/mL), trimethoprim (30 μg/mL), fosfomycin (30 μg/mL), colistin (20 μg/mL), daptomycin (4 μg/mL), rifampicin (62 μg/mL), gentamicin (8 μg/mL), chloramphenicol (30 μg/mL), ampicillin (100 μg/mL), doxycycline (100 mg/mL), amoxicillin (2,048 μg/mL), vancomycin (20 mg/mL), and tetracycline (15 mg/mL). In addition, a MIC estimation was established using E-test strips (BioMérieux^®^, France) for the following antibiotics: ampicillin, ciprofloxacin, fosfomycin, gentamicin, streptomycin, tobramycin, and ceftazidime.

### 2.5. Swimming test

An overnight culture was inoculated with a sterile toothpick on swimming plates. The swimming motility plates were prepared with 0.3% agar, 1.0% peptone (Acros Organics, Germany), and 0.5% NaCl (≥99.5% Carl Roth, Germany) (Zorzano et al., 2005).

### 2.6. Biofilm production test

The assay was conducted in 96-well microtiter dishes made of polypropylene (Greiner Bio One, Germany). Bacterial cells of overnight cultures at 30°C were adjusted to 10^7^ CFU/mL in LB Lennox medium (Carl Roth, Germany). Plates were inoculated with the bacterial suspensions (100 μl per well) and incubated at 30°C for 24 h, 48 h, and 72 h. Next, the top of the plate was washed with water and placed in a new plate with 100 μl of crystal violet (1%) (O'Toole and Kolter, 1998). The plates were incubated for 30 min at room temperature and rinsed thoroughly and repeatedly with water. Finally, the dye was solubilized in acetic acid 35% (100 μL per well). The absorbance was determined at 595 nm. Each result represents 40 independent replicates of the experiment. For analyzing differences in biofilm formation, a Kruskal–Wallis test was conducted using software R version 4.1.1 (2021-08-10) (R Development Core Team, 2015).

### 2.7. Sample preparation and liquid chromatography–label-free quantification mass spectrometry (LC–LFQ-MS)

*A. hydrophila* proteomic analysis was carried out on exponential growing cultures treated with different BAC concentrations (9.5 μg/mL, 19 μg/mL, 38 μg/mL) for 30 min at 30°C. The main aim of this experiment was to determine the global response of *A. hydrophila* INISA09 strain to BAC. Non-treated samples were used as control. Each experiment consisted of six replicates. After exposure, the bacterial cells were pelleted by centrifugation at 4°C, 10,000 x g for 2 min, the supernatant was discharged, and the pellet was kept at −80°C. The bacterial pellets were resuspended in 100 μL of urea denaturing buffer (6 M urea, 2 M thiourea, and 10 mM HEPES, pH 8.0) followed by 8 cycles of freezing–unfreezing. All further sample preparation steps were performed as previously published (Rodríguez-Rojas and Rolff, 2020). After the digestion of proteins into peptides, salts and contaminations (e.g., BAC) were removed before LC-MS analysis by SDB-RPS StageTips (Empore™ 2241) (Rappsilber et al., 2007). After elution from the StageTips, peptides were dried under a vacuum.

Peptides trifluoroacetic acid (TFA) were reconstituted in 30 μL of 0.05% trifluoroacetic acid (TFA), 2% acetonitrile in water, and 1 μL and TFA analyzed by a reversed-phase capillary nano liquid chromatography system (Ultimate 3000, Thermo Scientific) connected to a Q Exactive HF mass spectrometer (Thermo Scientific). Samples were injected and concentrated on a trap column (PepMap100 C18, 3 μm, 100 Å, 75 μm i.d.×2 cm, Thermo Scientific) equilibrated with 0.05% TFA in water. After switching the trap column inline, LC separations were performed on a capillary column (Acclaim PepMap100 C18, 2 μm, 100 Å, 75 μm i.d. ×25 cm, Thermo Scientific) at an eluent flow rate of 300 nL/min. Mobile phase A contained 0.1% formic acid in water, and mobile phase B had 0.1% formic acid in 80% acetonitrile / 20% water. The column was pre-equilibrated with 5% mobile phase B, and peptides were separated using a 5–44% mobile phase B gradient within 35 min. Mass spectra were acquired in a data-dependent mode utilizing a single MS survey scan (m/z 300–1650) with a resolution of 60,000 in the Orbitrap and MS/MS scans of the 15 most intense precursor ions with a resolution of 15,000. HCD fragmentation was performed for all peptide ions with charge states of 2+ to 5+ using normalized collision energy of 27 and an isolation window of 1.4 m/z. The dynamic exclusion time was set to 20 s. Automatic gain control (AGC) was set to 3 × 10^6^ for MS scans using a maximum injection time of 20 ms. For MS2 scans, the AGC target was set to 1 × 10^5^ with a maximum injection time of 25 ms.

### 2.8. LC-MS data processing and protein quantification

MS and MS/MS raw data were analyzed using the MaxQuant software package (version 2.0.3.0) with the implemented Andromeda peptide search engine (Tyanova et al., 2016a). For the database search, a FASTA formatted protein sequence database of *A. hydrophila* downloaded from Uniprot (Bateman et al., 2023) (4,121 proteins, taxonomy 380,703, 22 March 2022) was used. Filtering and statistical analysis were performed using Perseus software version 1.6.14 (Tyanova et al., 2016b) (Supplementary Table S3). Only proteins that were identified with LFQ intensity values in at least three out of six replicates (within at least one of the four experimental groups) were used for downstream analysis. Missing values were replaced from a normal distribution (imputation) using the default settings (width 0.3, downshift 1.8). Mean log2-fold differences between BAC-treated vs. non-treated control were calculated in Perseus, using Student's *t*-tests with permutation-based FDR of 0.05. Proteins are defined as significantly regulated if they express a *q*-value of < 0.05 (corresponds to the adjusted *p*-value) and at least a 2-fold change in intensity. The increased or decreased degree of overlapping proteins was represented as Venn diagrams using the online tool Venny (Oliveros, 2007–2015). Finally, the ontology and network of the identified proteins were retrieved with the Uniprot database (Bateman et al., 2023) and the STRING database (Szklarczyk et al., 2019) to obtain their functions and relations.

## 3. Results and discussion

**Figure 1:**
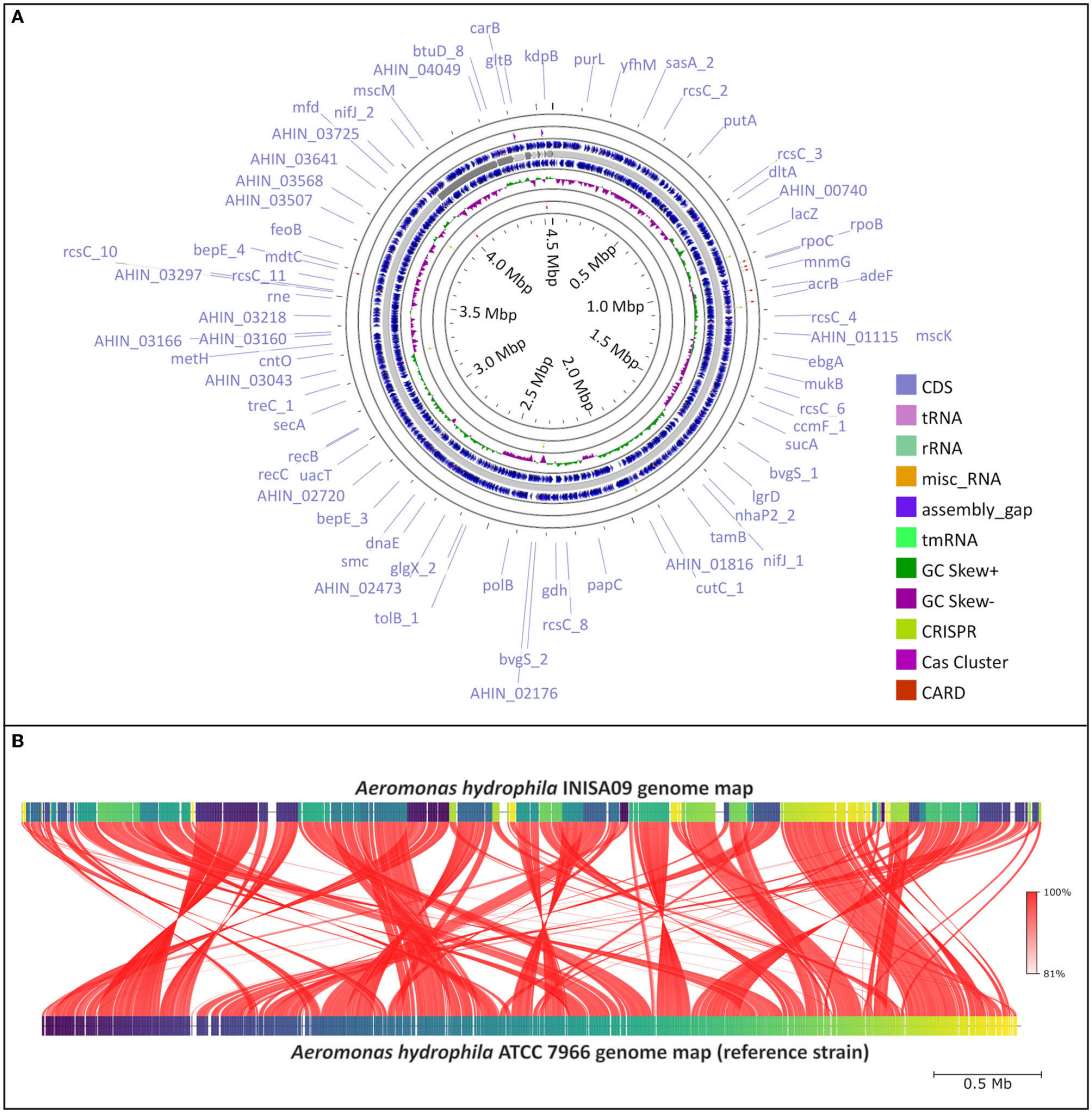
*Aeromonas hydrophila* INISA09 genome description. **(A)** Visualization of the genome performed using the CGView server (Stothard and Wishart, 2005), with CRISPRCasFinder (Couvin et al., 2018) and CARD (Alcock et al., 2019) modules for the visualization of CRISPR-Cas associated protein and antibiotic resistance genes, respectively. **(B)** Average nucleotide identity (ANI) comparison between *Aeromonas hydrophila* INISA09 and *Aeromonas hydrophila* ATCC 7966. The red lines represent the reciprocal mapping among both strains indicating the conservation degree.

**Figure 2:**
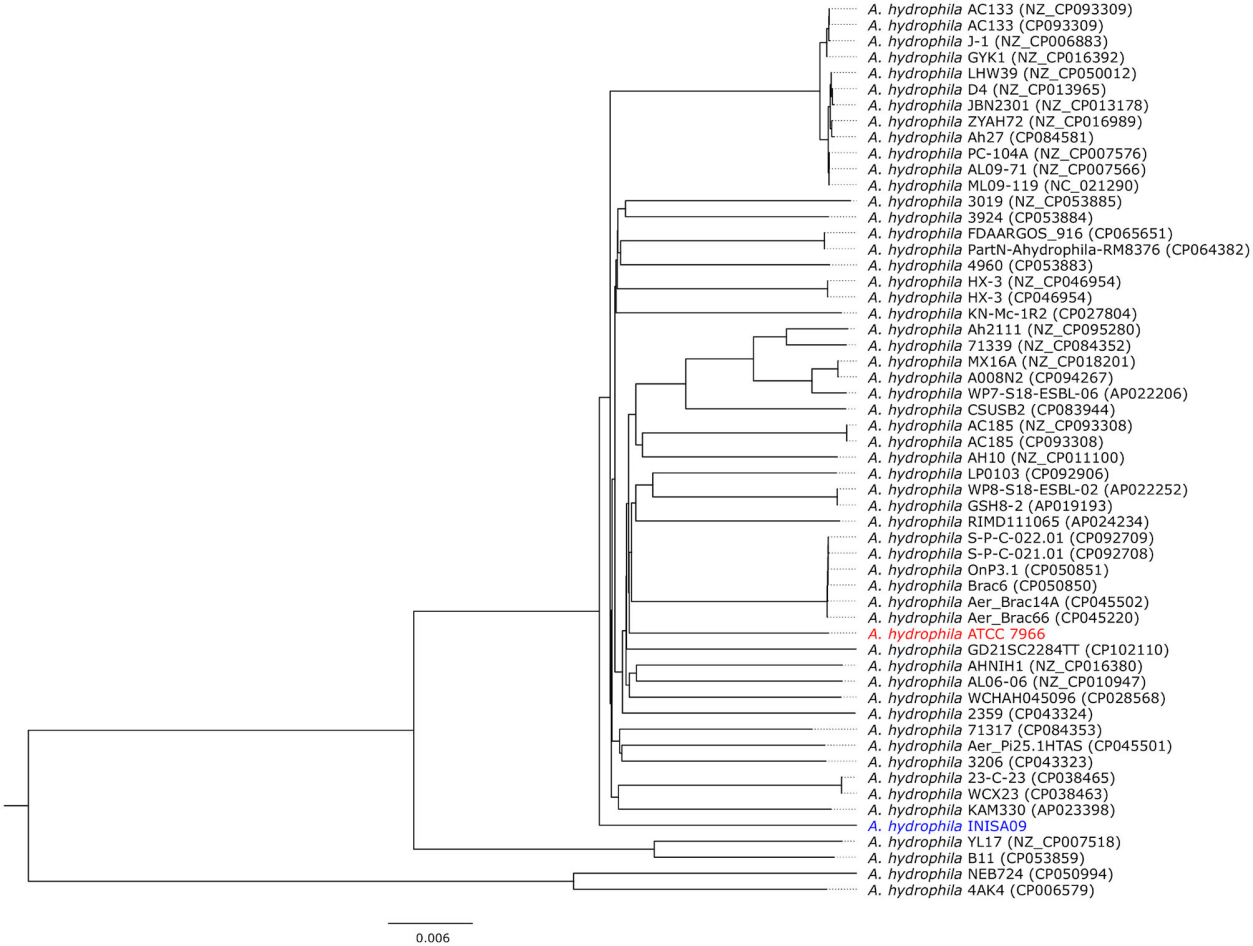
Phylogram representation of the divergence of 54 *Aeromonas hydrophila* strains. Details about the strains can be found in Supplementary Table S1 and Supplementary Figure S1. The ATCC 7966 (in red) and the INISA09 (in blue) are highlighted in the tree constructed from average nucleotide identity (ANI) comparison value (Jain et al., 2018) and visualized using FigTree v.1.4.4 (http://tree.bio.ed.ac.uk/software/figtree).

First, from an activated sludge previously exposed to BAC 10 mg/L (Chacón et al., 2021), a bacterial inoculum was transferred to TSA plates enriched to 25 mg/mL of BAC. After 24 h incubation at 28°C, only less-susceptible bacteria formed colonies. DNA extraction and *16S rRNA* gene sequencing were conducted by Macrogen^®^ (South Korea). Of the 20 different identified isolates, eight were identified as *A. hydrophila* with 99% of identity, while the others were *Serratia marcescens* (12), *Burkholderia cepacia* (1), and an unidentified bacteria. A preliminary susceptibility test showed that INISA09 had a low susceptibility to BAC (MIC = 38 μg/mL). Thereafter, we sequenced the bacterial genome of *A. hydrophila* INISA09 (NZ_JAPKVL000000000.1). The draft genome had approximately a size of 4.6 Mbp, 61.3% GC content, 121 tRNA genes, 14 rRNAs, 1 tmRNA, 50 miscRNAs, and 4,087 CDS. No CRISPR sequences or prophages were present in the genome (Figure 1A). These characteristics are in common with the reference strain *A. hydrophila* ATCC 7966 (Seshadri et al., 2006), which is recommended by European Committee on Antimicrobial Susceptibility Testing (EUCAST) as a reference strain in antibiotic susceptibility testing, and which was previously reported to be susceptible to BAC (Stratev and Vashin, 2014). The draft genome contained 4,273 genes. An analysis of single-nucleotide polymorphisms (SNPs) showed 106,503 mutations (compared to *A. hydrophila* ATCC 7966), with 16,099 mutations classified as non-synonymous mutations and 15,762 corresponding to missense variants. Some of these mutations were found in genes associated with transport, antimicrobial resistance, gene expression regulation, motility, and redox activity. The strain *A. hydrophila* INISA09 underwent a profound genome rearrangement and plasticity compared to other strains (Figures 1B, 2, and Supplementary material S1), probably fine-tuned by thousands of mutations (including compensatory mutations) for better survival in the heavily contaminated environment where the isolate was taken. The changes are so numerous that it is not possible to tackle the significance of each genomic using the classical microbial functional genetic approach. One possibility is that exposure to many different antimicrobials could contribute to accelerating the adaptation of the *A. hydrophila* INISA09. Many antibiotics increase the mutation rate in bacteria (Rodríguez-Rojas et al., 2013), and we recently showed that BAC also increases the mutation rate in some bacteria, such as *Escherichia coli* (Schmidt et al., 2022). Thus, this increased mutagenesis is a plausible explanation for the high number of SNPs observed in the genome of the *A. hydrophila* INISA09.

Phylogenetic analysis based on the *16S rRNA* gene sequences is inappropriate for closely related species or strains. This is because, in closely related species, the variable region of the *16S rRNA* gene does not provide sufficient variability to perform phylogenetic analysis accurately (Johnson et al., 2019; Nakano et al., 2022). Therefore, we also used whole-genome phylogeny to compare the divergence of *A. hydrophila* INISA09 with other 53 *A. hydrophila* strain genomes. The resulting phylogenetic tree (Figure 2) confirmed that *A. hydrophila* INISA09 is very dissimilar to at least 90% of the strains, indicating the drastic changes imposed by selective the environmental pressure. In addition, the divergence may include the absence of prophages, which is uncommon for this species (see Supplementary Table S1). During highly stressful conditions, prophages have been observed to be induced at a high frequency (Feiner et al., 2015). This induction can cause the lytic cycle, ultimately resulting in the death of the host. However, in a heavily antimicrobial-polluted environment, genetic variants lacking prophages may be selected for in order to increase the likelihood of survival. In the same vein, it has been observed that during the evolution of *E. coli* against increasing concentrations of hydrogen peroxide, another biocide, a high number of inactivating mutations were present in the prophage genomes (Rodríguez-Rojas et al., 2020), suggesting a positive selection of prophage-free variants in highly stressful conditions. This wipeout of prophages could have significant consequences for bacterial communities since prophages are essential players in bacterial diversity and ecology (Nadeem and Wahl, 2017).

While exploring the *A. hydrophila* INISA09 genome, focusing on genes with known roles in antimicrobial resistance, we found mutations in 112 loci associated with transport functions: 33 related to antimicrobial resistance and 33 related to integral membrane composition. In addition, we identified mutations in genes such as *bcr* (bicyclomycin resistance protein), *arnA* (bifunctional polymyxin resistance protein ArnA), *drrA* (daunorubicin/doxorubicin resistance ATP-binding protein DrrA), *fsr* (fosmidomycin resistance protein), *emrB, mdtE, mdtA, mdtH, mdtL, norM, mdlB* (all multidrug resistance proteins), and *qacC* (quaternary ammonium compound-resistance protein QacC). The mutations in genes associated with efflux pumps include *acrB, acrA, acrE*, and the outer membrane protein *tolC* (outer membrane protein), which is part of the AcrAB-TolC multidrug efflux pump complex (Tikhonova and Zgurskaya, 2004). These results are consistent with a previous study conducted in *Salmonella enterica* serovar Typhimurium, showing that the TolC protein connects with the AcrAB efflux pump system to expel BAC to the extracellular space (Guo et al., 2014).

We also found intragenic mutations in genes that code for membrane-related proteins such as the porins *ompA* (outer membrane protein A) and *ompW* (outer membrane protein W); both are also related to the adhesion process and biofilm formation (Maiti et al., 2012; Wang et al., 2022). The porins *ompD* (outer membrane protein D) and *ompN* (outer membrane protein N) also showed mutations in the INISA09 strain, which might confer low susceptibility to benzalkonium chloride (BAC) or other biocides since cationic surfactants target the outer membrane as the primary site (Gilbert and Moore, 2005). In a previous study in *Escherichia coli*, some of these genes were differentially expressed under BAC exposure in a transcriptomic assay (Forbes et al., 2019). On the other hand, an *in vitro* experiment in *E. coli* showed that BAC could induce tolerance mediated by the genes *lpxL* and *lpxM*. These genes code for myristoyl transferases responsible for the last acylation steps in lipid A synthesis (Nordholt et al., 2021). The wild *A. hydrophila* INISA09 genome presented 18 mutations in this gene; three were non-synonymous mutations: Y21H, M59I, and Y225H. However, these mutations differ from those observed in *E. coli:* A96E, M148R, L207I, A242E, and W298G.

We analyzed the antibiotic susceptibility of *A. hydrophila* INISA09 strain by E-test and disk diffusion assays. The MIC estimated by an E-test showed different degrees of susceptibility to seven antibiotics, including ampicillin >256 μg/mL, ciprofloxacin 0.064 μg/mL, fosfomycin 8 μg/mL, gentamycin 1 μg/mL, streptomycin 8 μg/mL, tobramycin 1.5 μg/Ml, and ceftazidime 0.032 μg/mL. A previous study conducted in environmental isolates of *Aeromonas* sp. (Goñi-Urriza et al., 2000) showed high resistance to penicillins such as ampicillin and increased susceptibility to the third generation of cephalosporins. Our results were similar for the susceptibility of *A. hydrophila* INISA09 to the third generation of cephalosporins, indicating the absence of extended-spectrum beta-lactamases. The values obtained in comparison with those of Goñi-Urriza et al. (2000) were the same for gentamycin (1 μg/mL) and were lower for streptomycin (16 μg/mL) and third-generation cephalosporins (cefotaxime 8 μg/mL). We highlight that strain INISA09 was highly susceptible to fosfomycin (0.2–8 μg/mL). We have included some antibiotics such as amoxicillin, ampicillin, and vancomycin to investigate whether resistance to BAC caused some collateral sensitivity as observed for resistance to other cationic drugs (Lázár et al., 2018).

The EUCAST (EUCAST, 2022) informed that all clinical isolates of *A. hydrophila* display intrinsic resistance to ampicillin, amoxicillin, amoxicillin–clavulanic acid, ampicillin–sulbactam, and cefoxitin. *A. hydrophila* is intrinsically resistant to benzylpenicillin, glycopeptides, lipoglycopeptides, fusidic acid, lincosamides, streptogramins, rifampicin, oxazolidines, and macrolides (except azithromycin). In this case, *A. hydrophila* INISA09 was only resistant to ampicillin (penicillin) and susceptible to ciprofloxacin (fluoroquinolone), tobramycin, streptomycin, gentamycin (aminoglycosides), ceftazidime (cephalosporin), and fosfomycin (epoxide). It is important to mention that the criteria of the European Center for Disease Prevention and Control (ECDC) define multidrug resistance (MDR) as acquired non-susceptibility to at least one agent in three or more antimicrobial categories (Magiorakos et al., 2012). Table 1 details the antibiotics tested and their classification according to the resistance level recommended by EUCAST for *Aeromonas sp*. and *Vibrio sp*. (another phylogenetically related Gammaproteobacterium), and in Supplementary Table S1, we show the tested concentration and inhibition halo diameter.

**Table 1:** Antibiotic susceptibility testing of *Aeromonas hydrophila* INISA09 determined by the disk diffusion method for different antibiotic families.

**Therapeutic class**	**Antimicrobial**	**Phenotype**
Aminoglycoside	Gentamicin	Susceptible
	Streptomycin	Susceptible^*^
Cephalosporine	Ceftazidime	Susceptible^*^
Dihydropyrimidine	Trimethoprim	Susceptible^*^
Epoxide	Fosfomycin	Susceptible
Glycopeptide	Vancomycin^*^	Intrinsic resistant
Lipopeptide	Daptomycin^*^	Intrinsic resistant
Penicillin	Amoxicillin^*^	Intrinsic resistant
	Ampicillin^*^	Intrinsic resistant
Phenicol	Chloramphenicol	Susceptible
Polymyxin	Colistin	Susceptible
Quinolone	Ciprofloxacin	Sensible
Rifamycin	Rifampicin^*^	Intrinsic resistant
Tetracycline	Doxycycline	Susceptible^*^
	Tetracycline	Susceptible^*^

* *Vibrio* sp. breakpoint according to EUCAST (EUCAST, 2022).

See Supplementary Table S2 for complete information such as inhibition zone diameters and tested concentrations.

We analyzed the effect of different near-MIC BAC concentrations on the growth curve of the INISA09 strain. To this end, we used the Growthcurver R package, which fits growth curve data to the standard form of the logistic equation commonly used in ecology and evolution (Sprouffske and Wagner, 2016), due to its limitations as low fitting in bacteria with very slow or fast doubling time since the first ones can produce high optical densities by extracellular products presence or low optical densities associated with a low growing rate; the parameters estimated are shown in Supplementary Figure S2. To this end, we employed the estimated MIC for BAC (38.0 μg/mL) to evaluate parameters related to growth, such as the growth rate (*r*-value), the maximum population size or carrying capacity (*k*-value), the generation time, and the empirical area under the curve (auc_e_) estimated from the obtained optical density (OD_600_) data. We compared these values with a non-treated control.

**Figure 3:**
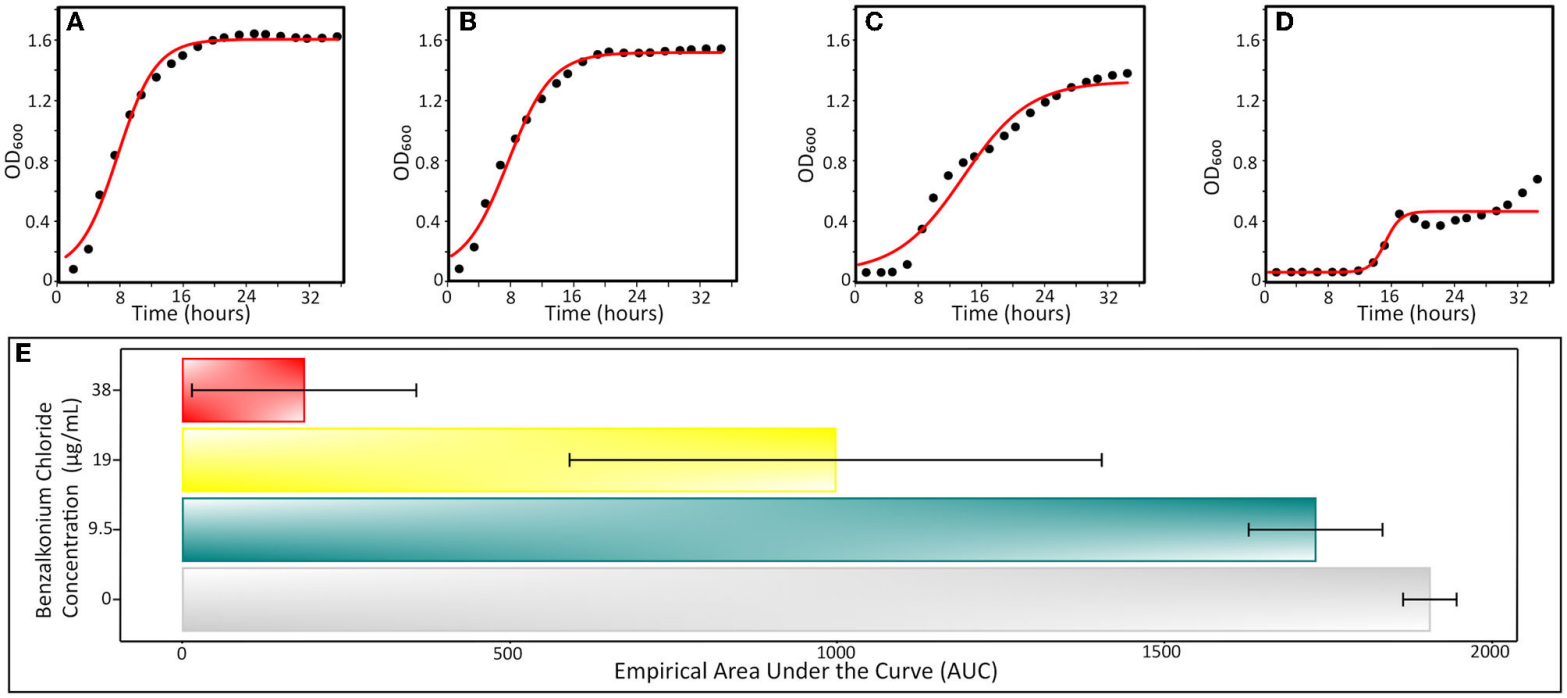
Typical growth curves of *Aeromonas hydrophila* INISA09 exposed to different BAC concentrations. **(A)** Non-treated control, **(B)** BAC 9.5 μg/mL, **(C)** BAC 19 μg/mL, **(D)** 38 μg/mL doses; and **(E)** empirical area under the curve, a global parameter that integrates other parameters such as growth rate, carrying capacity, death phase, and initial population size. The red line represents the logistic regression model obtained from the absorbance data. The empirical AUC was obtained from the experimental absorbance obtained curve from five independent repetitions according to the previously described procedure (Sprouffske and Wagner, 2016).

In Table 2, we present the results for relevant growth curve parameters (see also Supplementary Figure S2). We found significant statistical differences for the carrying capacity (*k*, which describes the maximum reachable biomass of the culture, *p* = 1.5 × 10^−7^) and the empirical area under the curve parameters (auc_e_, a global parameter that integrates effects on the initial population size, maximum growth rate, and carrying capacity, *p* = 7.7 × 10^−7^). In a dose–response curve analysis, the EC_50_ (half maximal effective concentration) of BAC for INISA09 corresponded to 33.0 μg/mL. Figure 3 shows a typical growth curve behavior of *A. hydrophila* under three different BAC concentrations: 9.5 μg/mL, 19 μg/mL, and 38 μg/mL, and the average area under the curve obtained. This strain's resistance level is notably higher than that of other bacteria, which were reported to have the following ecological cut-off concentrations based on the MIC-distributions of a total of 3327 microbial isolates: 128 μg/mL for *Salmonella* spp.; 64 μg/mL for *E. coli*; 32 μg/mL for *K. pneumoniae*; 32 μg/mL for *Enterobacter* spp.; 16 μg/mL for *S. aureus*; 8 μg/mL for *E. faecium*; and 8 μg/mL for *E. faecalis* (Morrissey et al., 2014).

**Table 2:** Parameters descriptive of growth for *Aeromonas hydrophila* INISA09 grown in the presence (38 μg/mL) and absence of BAC. See also Supplementary Figure S2.

**Parameter**	**BAC exposed**	**Non-BAC exposed**
*r*-value	1.8 x 10^−3^	6.2 x 10^−3^
*k*-value^*^	1.5 x 10^−3^	1.1
auc${\;}_{\text{e}}^{*}$	1.7	968
Generation time	877 min	111 min

* Statistical difference *p* ≤ 0.05.

Growthcurver R package fits the logistic equation of ecology and evolution (repeated OD_600_ measurements taken from a plate reader over time) to microbial growth curve data. From this fit, a variety of metrics are provided, including the maximum growth rate (*r*), the carrying capacity (*k*), and the area under the logistic curve (*AUC*) (Sprouffske and Wagner, 2016).

We also tested bacterial mobility using the swimming motility plate assay (Zorzano et al., 2005). *A. hydrophila* INISA09 showed a moderate swimming capacity (<5 cm in diameter of swimming). A biofilm formation assay shows a substantial increase in biofilm mass in the first 48 h (Supplementary Figure S3). The statistical analysis showed differences in biofilm production in the three tested periods (*p* = 1.1 x 10^−3^). However, *post-hoc* analyses indicated that the differences occur among the first 48 h (*p* = 0.03), whereas the difference between 48 and 72 h is not significant (*p* = 0.16). This result indicates that INISA 09 biofilms mature within 48 h under experimental conditions.

**Figure 4:**
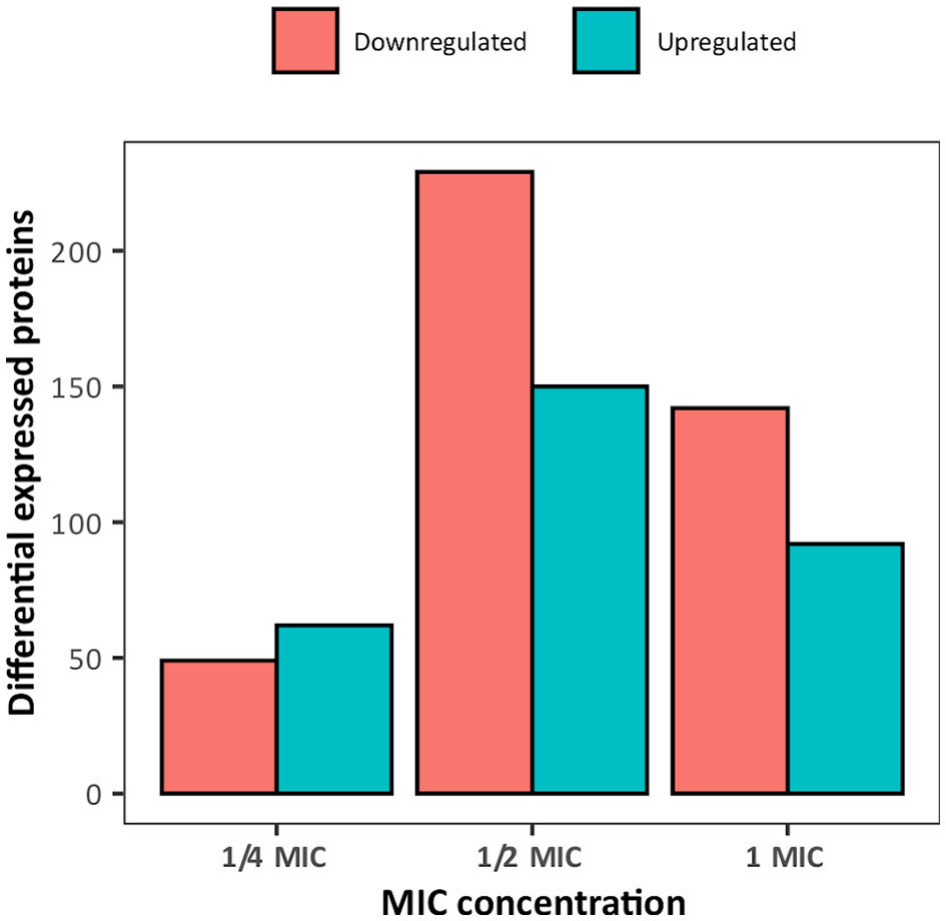
Overview of the number of differentially expressed proteins in *Aeromonas hydrophila* INSA09 after 30 min of exposure to BAC (¼ MIC: 9.5 μg/mL, ½ MIC: 19 μg/mL, and MIC: 38 μg/mL doses).

**Figure 5:**
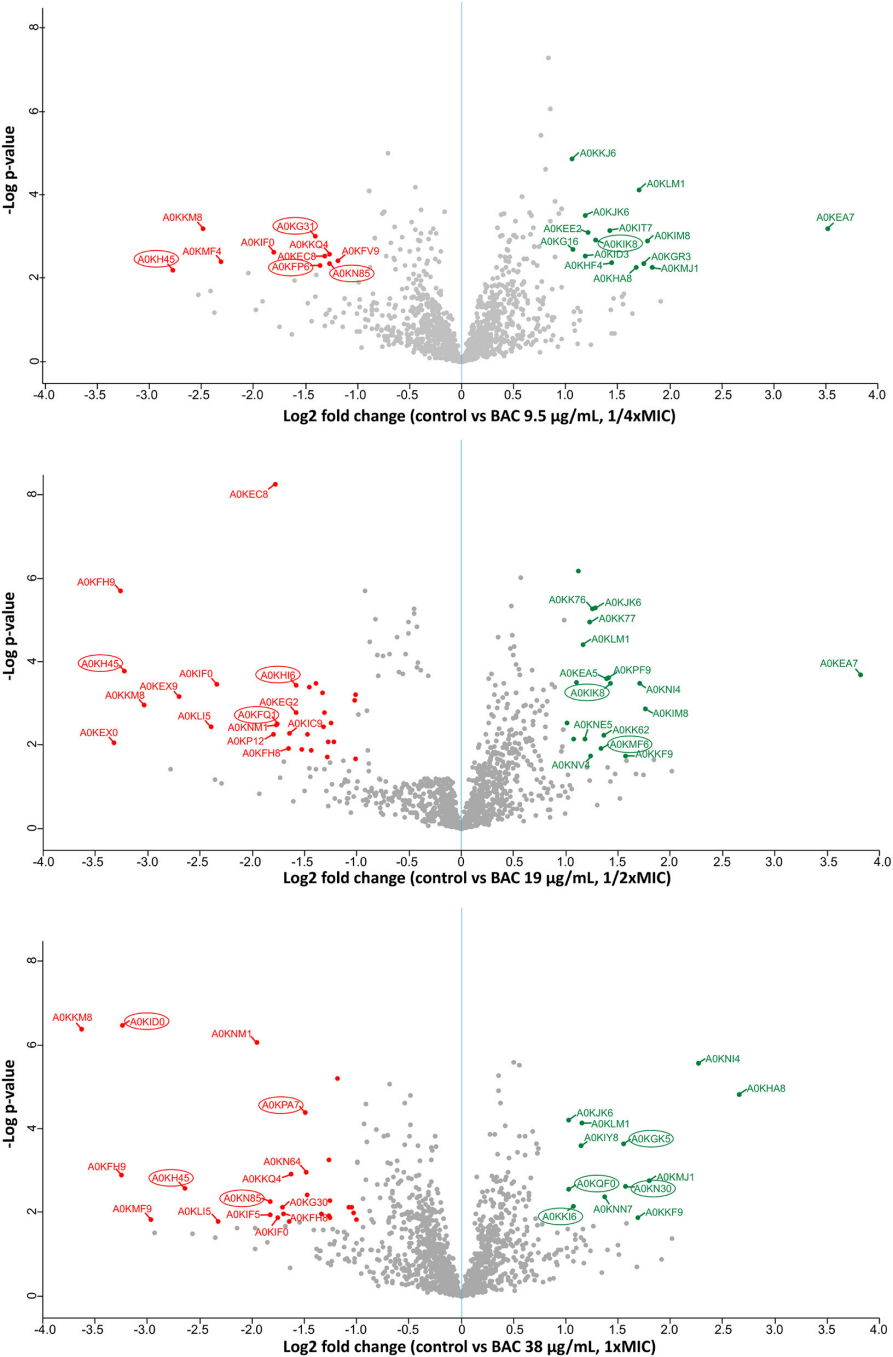
Label-free quantification LC-MS proteomics results. Volcano plot showing the log2 fold change of mean relative protein intensities of *Aeromonas hydrophila* determined by LC-MS and label-free quantification (BAC-treated vs. non-treated control). Cells have been treated with 9.5; 19; or 38 μg/mL BAC as indicated in the y-axis label. Proteins with significantly higher abundance in non-treated controls are colored in red (downregulated upon BAC treatment), while proteins with significantly higher abundance after BAC treatment are colored in green (upregulated). Proteins are defined as significantly regulated if they express a *q*-value < 0.05 (FDR adjusted *p*-value) and at least a 2-fold change in intensity. Proteins marked with a circle correspond to membrane-related proteins, according to the Uniprot database (Bateman et al., 2023).

To elucidate the mechanistic basis for the low susceptibility to BAC of the *A. hydrophila* INISA09, we performed a label-free quantification liquid chromatography–mass spectrometry proteomic analysis. To this end, we exposed the cells for 30 min to different concentrations of BAC and compared them to the non-treated control. The chosen BAC concentrations were 9.5, 19, and 38 μg/mL, corresponding to a quarter, half, and minimal inhibitory concentration; these experiments were carried out with a higher inoculum (~10^8^ CFU/mL) to have more than enough cells for the protein extraction. We found 111, 379, and 233 proteins with a significant change in relative abundance when the bacteria were treated with ¼ MIC (9.5 μg/mL), ½ MIC (19 μg/mL), and the MIC (38 μg/mL), respectively (Figure 4). Volcano plots representing the differential expression of all quantified proteins are shown in Figure 5. Tables 3, 4 list 15 proteins with the largest fold change in each experiment. The whole dataset is shown in Supplementary Table S3.

**Table 3:** Main downregulated proteins, measured by LC-MS using label-free quantification in *Aeromonas hydrophila* after exposure to three different BAC concentrations.

$\frac{1}{4}$ **MIC** μ**g/mL**				$\frac{1}{2}$ **MIC** μ**g/mL**				**MIC** μ**g/mL**			
**Identifier**	**Protein**	**Fold change**	***q*** **value**	**Identifier**	**Protein**	**Fold change**	***q*** **value**	**Identifier**	**Protein**	**Fold change**	***q*** **value**
A0KH45	Putative outer membrane porin	−2.774	0.043	A0KEX0	Peptide deformylase	−3.320	0.021	A0KKM8	DUF2987 domain-containing protein	−3.624	0.000
A0KKM8	DUF2987 domain-containing protein	−2.488	0.015	A0KFH9	Iron-sulfur cluster assembly protein CyaY	−3.255	0.000	A0KFH9	Iron-sulfur cluster assembly protein CyaY	−3.244	0.012
A0KMF4	HutD family protein	−2.309	0.030	A0KH45	Putative outer membrane porin	−3.220	0.001	A0KID0	Protease HtpX	−3.242	0.000
A0KIF0	Hydrogen peroxide-inducible genes activator	−1.803	0.023	A0KKM8	DUF2987 domain-containing protein	−3.038	0.006	A0KMF9	Response regulator with TPR repeat, putative	−2.964	0.049
A0KG31	Phosphatidylglycerol-prolipoprotein diacylglyceryl transferase	−1.407	0.015	A0KEX9	DUF1488 domain-containing protein	−2.701	0.004	A0KH45	Putative outermembrane porin	−2.638	0.017
A0KFP6	Nucleoside permease NupC	−1.363	0.034	A0KLI5	Aminotransferase, class I and II	−2.394	0.012	A0KLI5	Aminotransferase, class I and II	−2.328	0.017
A0KEC8	Sensory box/GGDEF family protein, putative	−1.314	0.026	A0KIF0	Hydrogen peroxide-inducible genes activator	−2.340	0.002	A0KNM1	3-oxoacyl-(Acyl-carrier-protein) reductase, putative	−1.953	0.001
A0KKQ4	Phosphoglycolate phosphatase, bacterial	−1.274	0.025	A0KP12	2-hydroxyglutaryl-CoA dehydratase, D-component	−1.797	0.016	A0KIF5	tRNA U34 carboxymethyltransferase	−1.828	0.043
A0KN85	Apolipoprotein N-acyltransferase	−1.269	0.032	A0KEC8	Sensory box/GGDEF family protein, putative	−1.777	0.000	A0KN85	Apolipoprotein N-acyltransferase	−1.826	0.027
A0KFV9	Maltose O-acetyltransferase	−1.185	0.029	A0KNM1	3-oxoacyl-(Acyl-carrier-protein) reductase, putative	−1.765	0.010	A0KIF0	Hydrogen peroxide-inducible genes activator	−1.754	0.047
A0KIC9	ATP-dependent dethiobiotin synthetase BioD	−0.886	0.009	A0KFQ1	Transporter, CorA-like family	−1.761	0.010	A0KG30	Phosphoenolpyruvate-protein phosphotransferase	−1.707	0.032
A0KM14	Phosphate-specific transport system accessory protein PhoU	−0.876	0.039	A0KFH8	Carboxylesterase 2	−1.648	0.027	A0KFH8	Carboxylesterase 2	−1.695	0.041
A0KEP0	PTS system, mannitol-specific enzyme II, B component	−0.831	0.015	A0KIC9	ATP-dependent dethiobiotin synthetase BioD	−1.646	0.015	A0KKQ4	Phosphoglycolate phosphatase. bacterial	−1.625	0.013
A0KM49	Negative regulator of flagellin synthesis	−0.765	0.012	A0KHI6	Serine transporter family protein	−1.583	0.00-	A0KPA7	TIGR04219 family outer membrane beta-barrel protein	−1.491	0.002
A0KQX0	Glutamine–fructose-6-phosphate aminotransferase	−0.740	0.014	A0KEG2	Deoxyribonuclease TatD	−1.581	0.008	A0KN64	ATP-dependent Zn proteases	−1.480	0.012

A *q*-value of < 0.05 corresponds to adjusted *p*-values after false discovery rate control (FDR).

**Table 4:** Main upregulated proteins, measured by LC-MS using label-free quantification, in *Aeromonas hydrophila* after exposure to three different BAC concentrations.

$\frac{1}{4}$ **MIC** μ**g/mL**				$\frac{1}{2}$ **MIC** μ**g/mL**				**MIC** μ**g/mL**			
**Identifier**	**Protein**	**Fold change**	***q*** **value**	**Identifier**	**Protein**	**Fold change**	***q*** **value**	**Identifier**	**Protein**	**Fold change**	***q*** **value**
A0KEA7	16 kDa heat shock protein A	3.523	0.015	A0KEA7	16 kDa heat shock protein A	3.821	0.001	A0KHA8	HcpA homolog	2.656	0.002
A0KMJ1	Usp domain-containing protein	1.830	0.039	A0KIM8	Molybdopterin synthase sulfur carrier subunit	1.762	0.006	A0KNI4	Biotin carboxyl carrier protein of acetyl-CoA carboxylase	2.268	0.001
A0KIM8	Molybdopterin synthase sulfur carrier subunit	1.788	0.015	A0KNI4	Biotin carboxyl carrier protein of acetyl-CoA carboxylase	1.706	0.002	A0KMJ1	Usp domain-containing protein	1.798	0.013
A0KGR3	Hemerythrin domain-containing protein	1.749	0.032	A0KKF9	dCTP deaminase	1.570	0.038	A0KKF9	dCTP deaminase	1.691	0.048
A0KLM1	4-hydroxyphenylpyruvate dioxygenase	1.707	0.010	A0KIK8	Acyl-coenzyme A dehydrogenase	1.429	0.002	A0KN30	Type IV pilus secretin PilQ	1.576	0.016
A0KHA8	HcpA homolog	1.677	0.039	A0KPF9	Translational regulator CsrA	1.410	0.002	A0KGK5	Outer membrane porin protein	1.555	0.004
A0KHF4	RNA pseudouridine synthase family protein	1.442	0.030	A0KEA5	16 kDa heat shock protein A	1.388	0.002	A0KNN7	Lipoprotein	1.370	0.023
A0KIT7	Glycerol-3-phosphate dehydrogenase	1.423	0.015	A0KK62	Pyrimidine/purine nucleoside phosphorylase	1.366	0.016	A0KLM1	4-hydroxyphenylpyruvate dioxygenase	1.158	0.002
A0KIK8	Acyl-coenzyme A dehydrogenase	1.285	0.015	A0KMF6	Azurin	1.334	0.027	A0KIY8	Motility-associated factor glycosyltransferase family protein	1.143	0.004
A0KEE2	N5-carboxyaminoimidazole ribonucleotide mutase	1.217	0.015	A0KJK6	Succinate dehydrogenase iron-sulfur subunit	1.286	0.000	A0KKI6	Paraquat-inducible protein B	1.076	0.023
A0KID3	Pyridine nucleotide-disulfide oxidoreductase family protein	1.191	0.027	A0KK76	3-ketoacyl-CoA thiolase	1.251	0.000	A0KQF0	Sodium: dicarboxylate symporter family	1.031	0.002
A0KJK6	Succinate dehydrogenase iron-sulfur subunit	1.188	0.011	A0KNV4	DUF469 domain-containing protein	1.241	0.038	A0KJK6	Succinate dehydrogenase iron-sulfur subunit	1.027	0.004
A0KG16	Malate dehydrogenase	1.070	0.022	A0KK77	Fatty oxidation complex. alpha subunit FadJ	1.227	0.001	A0KGC5	Methyl-accepting chemotaxis protein	0.740	0.0032
A0KKJ6	PhaF	1.066	0.006	A0KNE5	Ribosome maturation factor RimP	1.180	0.019	A0KPY7	Stringent starvation protein B	0.729	0.018
A0KPW7	UDP-3-O-acyl-N-acetylglucosamine deacetylase	0.958	0.014	A0KLM1	4-hydroxyphenylpyruvate dioxygenase	1.162	0.001	A0KG36	30S ribosomal protein S20	0.0718	0.004

A *q*-value of < 0.05 corresponds to adjusted *p*-values after false discovery rate control (FDR).

**Figure 6:**
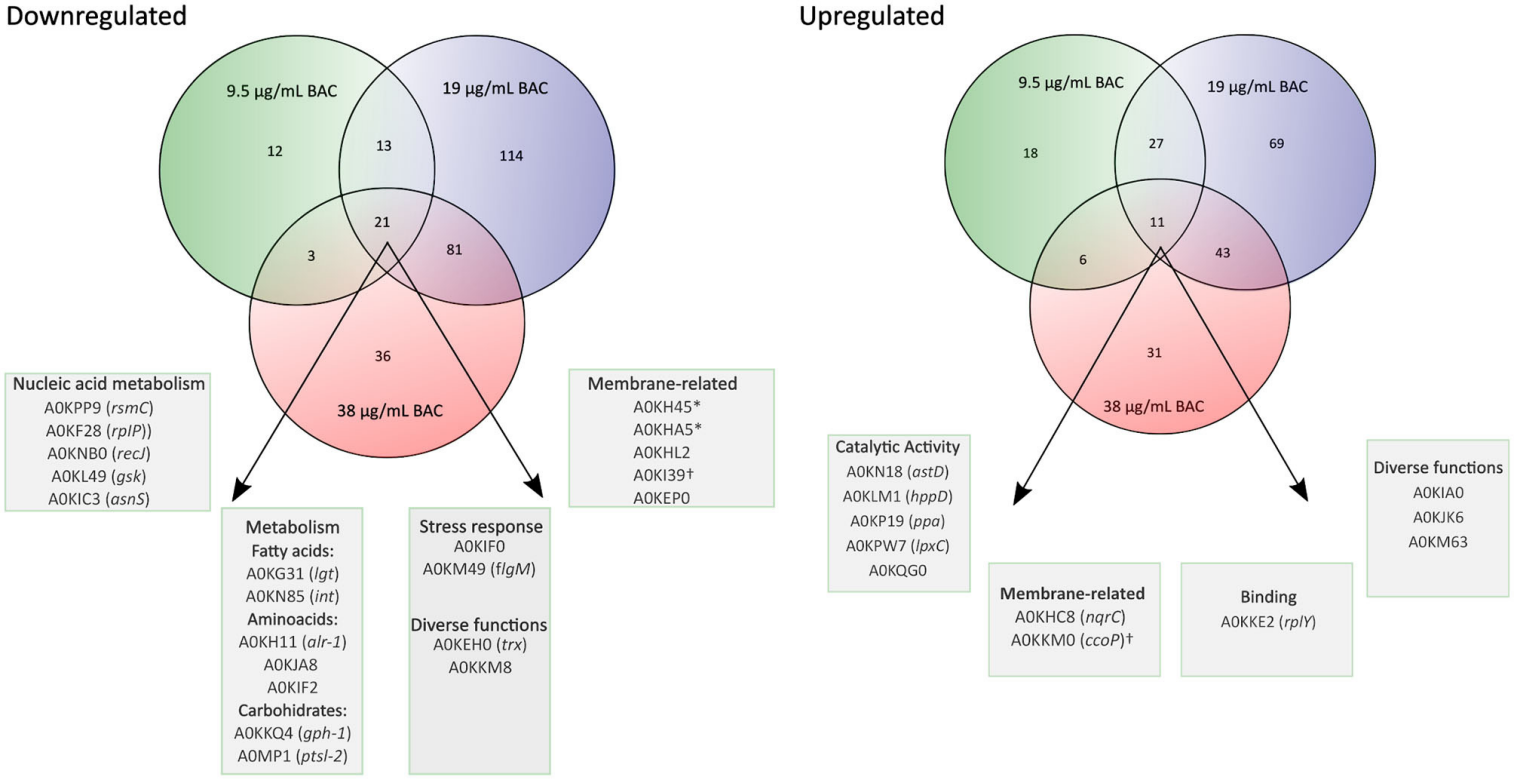
Venn diagrams showing the overlap of proteins that change significantly in their relative abundance (BAC-treated vs. non-treated control) at different BAC concentrations (¼ MIC −9.5 μg/mL-, ½ MIC−19 μg/mL-, and 1 MIC −38 μg/mL-). The porins are marked with an asterisk (*), and the cytochromes are marked with the symbol †.

In Figure 6, we show a Venn diagram illustrating the common core set of protein expressions and the dissimilarities among the three used BAC concentrations. A total of 21 downregulated proteins upon BAC treatment consistently coincide across all three conditions. These include five envelope-related proteins, including the membrane proteins A0KH45, A0KHA5, and A0KI39, the cell wall degradation protein A0KHL2, and a carbohydrate-associated transporter A0KEP0. Two of these proteins are related to different stress responses: A0KIF0 is an activator of hydrogen peroxide-inducible genes, and A0KM49 is a negative regulator of flagellin synthesis. Another 12 expressed genes are responsible for metabolic pathways, such as nucleic acid and fatty acid metabolism. One is a disulfide-reductase enzyme (A0KEH0), and the other is an undetermined function protein (DUF-domain protein) (A0KKM8). In addition, the upregulated proteins include six enzymes associated with metabolic processes (A0KN18, A0KLM1, A0KP19, A0KPW7, and A0KQG0), two membrane-related proteins (A0KHC8 and A0KKM0, both with oxidoreductase activity associated with electron transport), and three proteins with binding functions (A0KKE2 to ribosomes, and A0KIA0 and A0KJK6 to ion reductase enzymes). The last one, A0KM63, is a DUF-domain protein with an unknown function.

The results of the quantitative proteome analysis indicate that the basal response of *A. hydrophila* INISA09 to BAC exposure includes membrane rearrangements and stabilization of the oxidative cell status. Although there are few examples of environmental isolates exposed to BAC for comparative analysis, these results are consistent with those previously reported in a transcriptomic study of *Escherichia coli*. In this study, exposure to BAC reduced the expression of outer membrane porins, decreased motility and chemotaxis, and increased the expression of respiration-associated genes (Forbes et al., 2019).

A closer examination of the proteins with the highest differential expression level (Tables 3, 4) reveals that three proteins are strongly downregulated across all three tested concentrations. The protein A0KH45 is described as a putative outer membrane porin and a member of the OprD family. This group of proteins is associated with the transport of essential amino acids, and mutations in its sequence can lead to carbapenem resistance (Bateman et al., 2023). A previous study reported that reduced levels of its expression in the outer membrane could allow carbapenem accumulation in outer membrane vesicles and reduce free carbapenems within the cell (Chevalier et al., 2017). Lowering the expression of porins, such as OmpC, OmpD, and OmpF, is one mechanism that decreases the susceptibility of Gram-negative bacteria to biocides (Ferenci and Phan, 2015). In this regard, the INISA09 strain presented mutations in ompD. It has been previously described that *A. hydrophila* downregulates porin expression under iron-restricted conditions (Wang et al., 2017). *A. hydrophila* may also use this mechanism to resist stress conditions, such as biocide exposure or starvation.

The protein A0KKM8 appears in the databases uncharacterized, containing a DUF2987 domain. According to the GenBank database, this domain is present in various *Vibrio* species, including *V. parahaemolyticus, V. nigripulchritudo, V. natriegens, V. cholerae, V. cyclitrophicus, V. antiquaries, V. lentus, V. alginolyticus, and V. mimicus*, as well as in other species such as *Pseudoalteromonas rubia, Alteromonas macleodii, Shewanella baltica*, and *Alteromonas mediterranea*. It is also present in several *Aeromonas* species, including *A. caviae, A. dhakensis, A. jandei, A. media, A. salmonicida, A. veronii, A. sobria*, and *A. enteropelogenes*. A protein–protein interaction analysis using the STRING database (Szklarczyk et al., 2019) reveals a co-occurrence with molecules such as BamC, a lipoprotein part of the Outer Membrane Protein Assembly Complex involved in the assembly and insertion of beta-barrel structures into the outer membrane, and some uncharacterized proteins. This protein has not been characterized in detail until now. However, it is likely that the membrane-related protein is common in aquatic bacteria and is necessary for responding to stress. Therefore, we recommend studying the role of the protein A0KKM8 in future research *via* functional genomics to improve our understanding of its function, especially in biocide survival conditions.

The A0KIF0 protein, also known as hydrogen peroxide-inducible gene activator, was downregulated in the presence of BAC. This protein has been described as part of the OxyR regulon since 1990, and its function is to defend the cell against oxidative damage caused by hydrogen peroxide (Storz et al., 1990). In *A. hydrophila*, the expression of OxyR can be modulated by the LahS transcriptional regulator. Under stress conditions, LahS diminishes OxyR expression and enhances biofilm formation (Dong et al., 2018). A similar regulation may occur in INISA09, which suggests that the stress response to BAC is not mediated by hydrogen peroxide-related genes.

Additionally, the protein A0KKM49 (FlgM) is also downregulated. This response under stress conditions has been observed previously in *Salmonella* spp. where it was shown that damages in the flagella and membrane integrity could repress nutrient acquisition and activate the cell envelope stress response due to environmental factors affecting the bacterial envelop (Spöring et al., 2018). A similar mechanism is likely activated in *A. hydrophila* under BAC exposure.

For moderate and high doses of BAC, downregulation of several membrane proteins was observed, including A0KFH9 (iron–sulfur cluster assembly protein CyaY), which is related to the electron transport chain, radical generation, and regulation of biosynthesis of sulfur derivatives (Iannuzzi et al., 2011) and transport proteins such as A0KFQ1 (a member of the CorA-like family of transporters with metal ion transporter activity), A0KID0 (with metalloendopeptidase activity and zinc ion binding), and A0KPA7 (an outer membrane protein). In addition, some proteins related to fatty acid syntheses, such as A0KN85, A0KFH8, and A0KNM1, were also downregulated, indicating bacterial membrane reorganization and an alternative response to oxidative stress as a consequence of BAC exposure.

In the INISA09 strain, several cytochrome-related and metal-binding proteins were upregulated during BAC treatment, including A0KIM8, A0KHC8, and A0KKM0. Cytochromes and respiratory chain proteins can stabilize the cell's redox status without metal loss through an independent pathway of OxyR regulation (Mishra and Imlay, 2012). These results suggest that in response to BAC, membrane proteins, such as those involved in oxidative stress response, could be upregulated in the strain INISA09.

Under BAC stress conditions, several proteins related to fatty acid metabolism and membrane proteins were upregulated in the INISA09 strain. A0KK76 (FadI) and A0KK77 (FadJ) are two proteins regulated by the operon CO03404902 (Okuda and Yoshizawa, 2011), and both are part of the fatty acid oxidation with acetyl-CoA. According to STRING (Szklarczyk et al., 2019), these proteins interact with protein NuoC (A0KJ65), a membrane protein associated with electron transport across the membrane to conserve the redox energy in a proton gradient. Another upregulated protein observed that interacts with NuoC is A0KJK6 (the iron–sulfur subunit of succinate dehydrogenase), which is also regulated by the operon CO006649937 that regulates the expression of the succinate dehydrogenase-related genes (dhC, AHA_1924, sdhA, and AHA_1926). The overexpressed protein A0KNI4 (AccB) is part of an operon composed of the genes aroQ, accB, and accC, which are part of the acetyl coenzyme A carboxylase complex and involved in the fatty acid synthesis pathways.

Two other upregulated proteins, namely NifJ-1 and NifJ-2, are oxidoreductases that transfer electrons from pyruvate to flavodoxin and have iron centers. They interact directly with FadI, FadJ, AccB, A0KJK6, AstD (A0KN18), Mdh (A0KG16), and A0KIA0, all of which are related to oxidoreductase activities and fatty acid metabolism. This pattern is remarkable because most of the proteins mentioned have metal centers and can participate in electron transfer processes as mechanisms to balance oxidative stress disequilibrium (Crack et al., 2012). In case of the INISA09 strain, the disturbance caused by BAC at the membrane level could be managed with a reorganization of the fatty acid composition since the primary redox reactions are related to fatty acid metabolism, using redox reactions to equilibrate the oxidative stress.

Finally, we found correspondences between the most critically differentially expressed proteins (Tables 3, 4) and the presence of missense SNPs (see Table 5) (stop codons, deletions, and insertions with frameshift changes were not found). The average number of missense SNPs is 3 per differentially expressed protein. However, it is remarkable that the downregulated proteins A0KH45 (porin), A0KHI6 (transporter), and A0KMF4 (HUT domain protein) present more than 10 missense SNPs. Regarding upregulated proteins, A0KKE3 (histidine kinase) and A0KNN7 (lipoprotein) present more than 15 missense mutations in their sequence. However, further research is required to determine the effect of these mutations on the global BAC response presented by INISA09 or other environmental bacteria.

**Table 5:** Differentially expressed proteins and missense SNP present in *Aeromonas hydrophila* INISA09.

**Downregulated**			**Upregulated**		
**Protein**	**Gene**	**Number of missense SNPs**	**Protein**	**Gene**	**Number of missense SNPs**
A0KEC8	AHA_0044	3	A0KEA5	AHA_0007	1
A0KEC8	AHA_0044	3	A0KEA7	AHA_0008	1
A0KEG2	*tatD*	4	A0KEE2	*purE-1*	1
A0KEH0	*trx*	0	A0KG16	*mdh*	2
A0KEP0	AHA_0172	0	A0KG36	*rpsT*	0
A0KEX0	*def-1*	1	A0KGC5	AHA_0773	0
A0KEX9	AHA_0267	3	A0KGK5	AHA_0854	0
A0KF28	*rplP*	0	A0KGR3	AHA_0916	4
A0KFH8	AHA_0471	5	A0KHA8	AHA_1118	0
A0KFH9	*cyaY*	1	A0KHC8	*nqrC*	0
A0KFP6	AHA_0539	0	A0KHF4	AHA_1164	1
A0KFQ1	AHA_0544	3	A0KIA0	AHA_1463	5
A0KFV9	AHA_0602	1	A0KID3	AHA_1493	9
A0KG30	*ptsI-1*	0	A0KIK8	AHA_1573	8
A0KG31	*lgt*	0	A0KIM8	*moaD*	3
A0KH11	*alr-1*	2	A0KIT7	AHA_1652	2
A0KH45	AHA_1053	13	A0KIY8	AHA_1703	5
A0KHA5	AHA_1114	3	A0KJK6	AHA_1926	6
A0KHI6	AHA_1196	10	A0KK62	*ppnP*	2
A0KHL2	AHA_1222	5	A0KK76	*fadI*	2
A0KI39	AHA_1401	1	A0KK77	*fadJ*	5
A0KIC3	*asnS*	6	A0KKE3	AHA_2220	15
A0KIC9	*bioD*	1	A0KKF9	*dcd*	0
A0KID0	*htpX*	0	A0KKI6	AHA_2263	0
A0KIF0	*oxyR*	6	A0KKJ6	AHA_2274	0
A0KIF2	AHA_1515	0	A0KKM0	*ccoP*	1
A0KIF5	*cmoB*	6	A0KLM1	*hppD*	2
A0KJA8	AHA_1824	2	A0KM63	AHA_2860	9
A0KKM8	AHA_2306	2	A0KMF6	*azu*	1
A0KKQ4	*gph-1*	1	A0KMJ1	AHA_2988	1
A0KL49	*gsk*	0	A0KN18	*astD*	4
A0KLI5	AHA_2607	0	A0KN30	*pilQ*	5
A0KM14	*phoU*	0	A0KNE5	*rimP*	0
A0KM49	*flgM*	0	A0KNI4	*accB*	1
A0KMF4	AHA_2951	10	A0KNN7	AHA_3399	16
A0KMF9	AHA_2956	1	A0KNV4	AHA_3481	0
A0KN64	AHA_3224	3	A0KPF9	*csrA*	0
A0KN85	*lnt*	4	A0KPW7	*lpxC*	0
A0KNB0	*recJ*	6	A0KPY7	*sspB*	0
A0KNM1	AHA_3383	4	A0KQF0	AHA_4076	2
A0KP12	AHA_3543	6	A0KQG0	*pyrB*	0
A0KPA7	AHA_3652	1	A0KP19	*ppa*	0
A0KPP9	*rsmC*	10			
A0KQX0	*glmS*	9			

## 4. Conclusion

**Figure 7:**
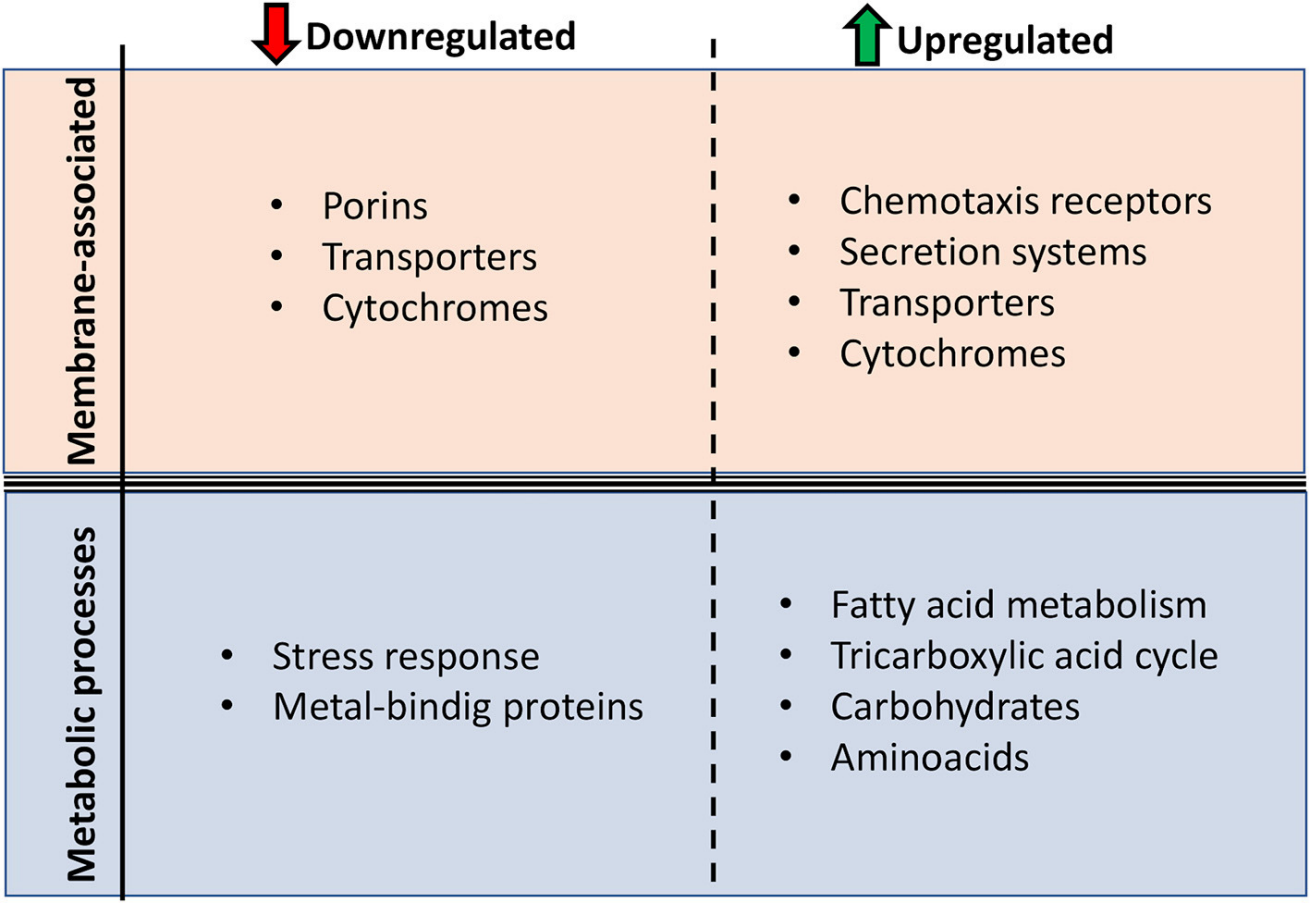
Main protein function changes presented by *Aeromonas hydrophila* INISA09 upon BAC exposure. The changes were divided into two levels: membrane composition and metabolic processes.

In summary, we have characterized the strain *A. hydrophila* INISA09 and its low susceptibility to BAC using various genomic, proteomic, and phenotypic techniques. This natural isolate from a Costa Rica domestic activated sludge underwent a profound genome rearrangement compared to other strains, probably fine-tuned by thousands of mutations for better survival in the heavily contaminated environment. The low susceptibility of *A. hydrophila* INISA09 to BAC can be related to changes in genes and proteins expression from the outer membrane, transmembrane transport, and fatty acid synthesis metabolic pathways (Figure 7). However, we recommend studying putative proteins such as A0KKM8, A0KH45, A0KGK5, A0KKI6, and A0KNV4 in future research using functional genomics strategies to identify or verify their function in biocide survival conditions. Finally, we provide information on mechanisms used by an environmental multidrug-resistant microorganism exposed to increasing amounts of substances such as disinfectants and other antimicrobials discharged into aquatic systems.

## Data availability statement

The datasets presented in this study can be found in online repositories. The names of the repository/repositories and accession number(s) can be found below: https://www.ncbi.nlm.nih.gov/nuccore/NZ_JAPKVL000000000.1.

## Author contributions

LC, FS, KR-J, and AR-R contributed to the conception and design of the study. LC organized the database. LC, EG-T, and BK performed the data analysis and wrote sections of the manuscript. BK performed the mass spectrometric measurements. LC, KR-J, and AR-R wrote the first draft of the manuscript. All authors contributed to the manuscript's revision, read, and approved the submitted version.
