# Supplementary material for: Mechanisms of low susceptibility to the disinfectant benzalkonium chloride in a multidrug-resistant environmental isolate of Aeromonas hydrophila
Source: Front Microbiol. 2023 Jun 2;14:1180128. doi: 10.3389/fmicb.2023.1180128 (PMC10272739; doi:10.3389/fmicb.2023.1180128)
Supplement: Supplementary file 1 [file Data_Sheet_1.zip › Supplementary_material.docx]

Supplementary Material

**Mechanisms of low susceptibility to the disinfectant benzalkonium chloride in a multidrug-resistant environmental isolate of *Aeromonas hydrophila***

**Luz Chacón, Benno Kuropka, Enrique González-Tortuero, Frank Schreiber, Keilor Rojas-Jiménez*, and Alexandro Rodríguez-Rojas***

**Correspondence:** Corresponding Author: Keilor Rojas-Jiménez: [keilor.rojas@ucr.ac.cr](mailto:keilor.rojas@ucr.ac.cr) / Alexandro Rodríguez-Rojas: [a.rojas@fu-berlin.de](mailto:a.rojas@fu-berlin.de), Alexandro.Rojas@vetmeduni.ac.at

## Supplementary Figures


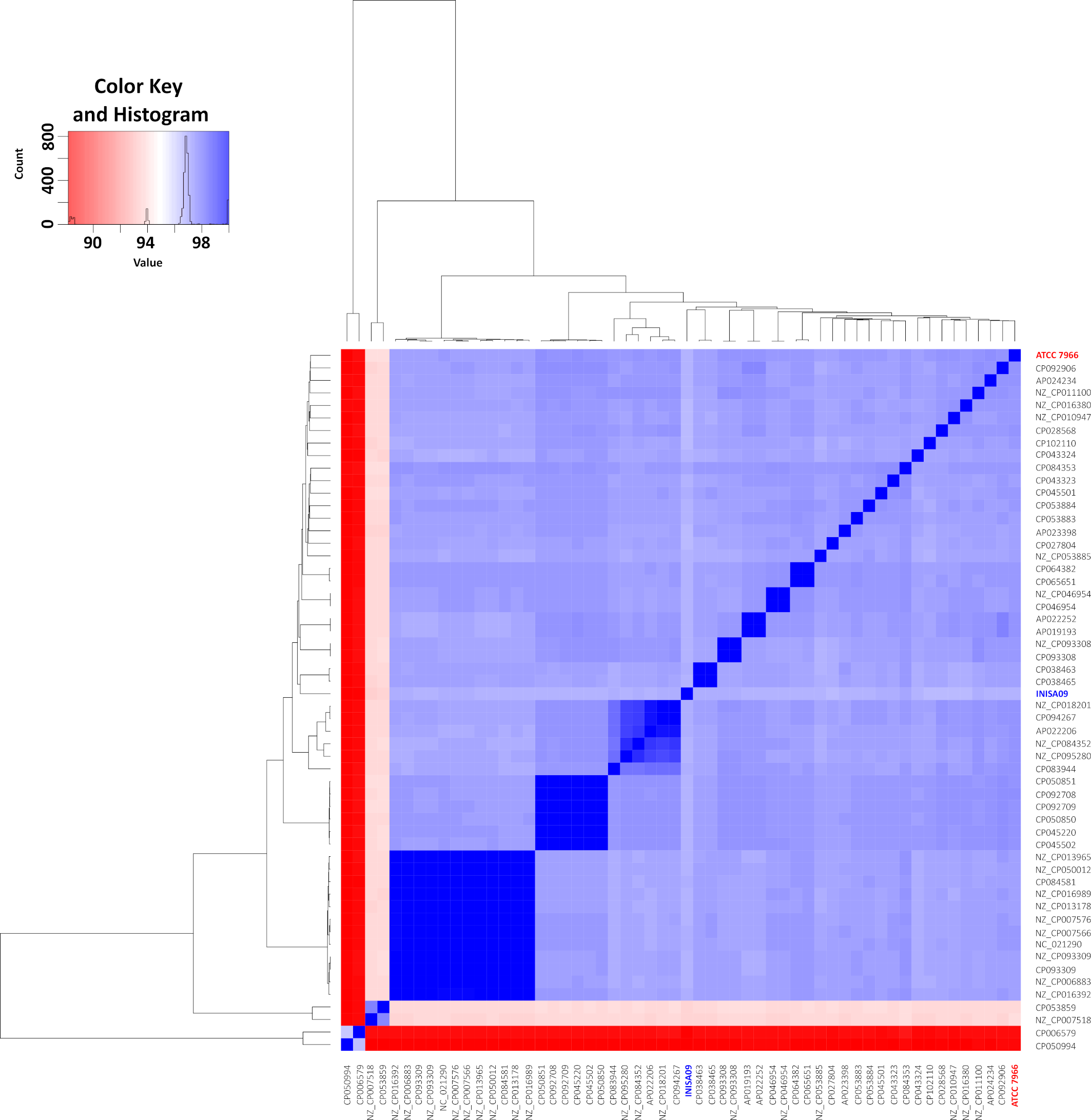


**Supplementary Figure 1.** Heatmap obtained from the Average Nucleotide Identity (ANI) (Jain et al., 2018) comparison obtained from 54 *Aeromonas hydrophila* strains: 52 detailed in Table S1, ATCC 7966 strain, and INISA09 strain.


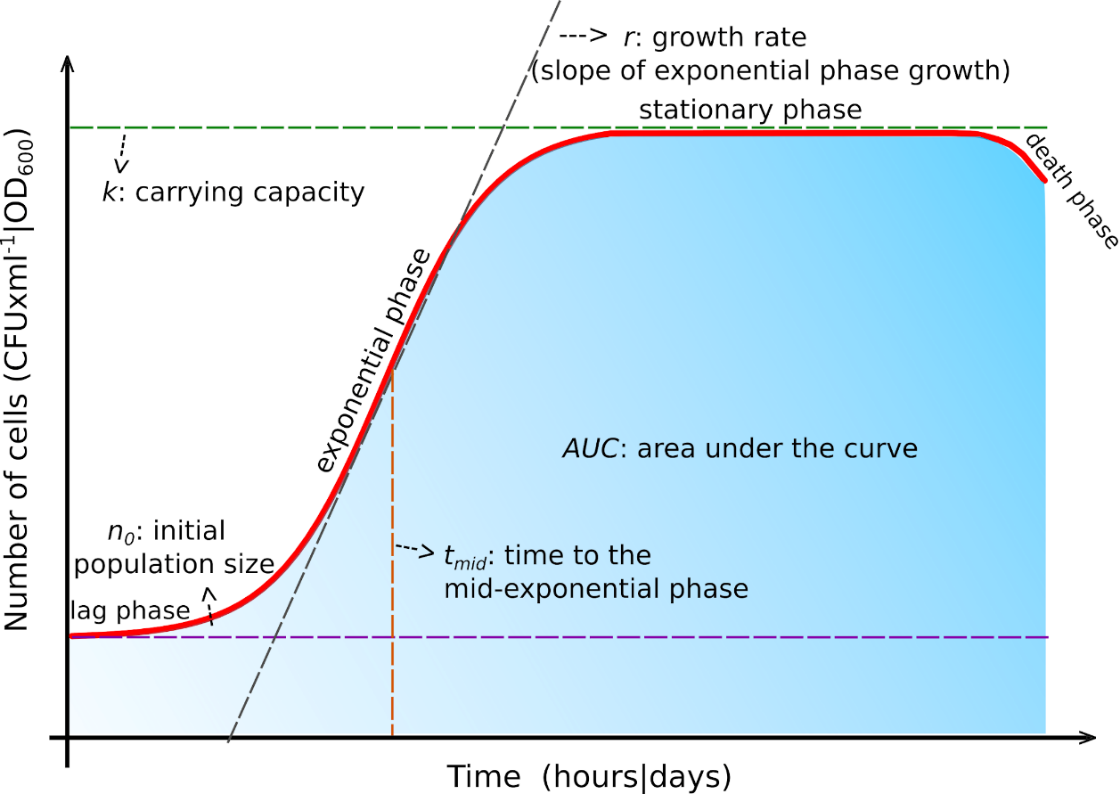


**Supplementary Figure 2.** Growth curve parameters analyzed by the Growthcurver R package (Sprouffske and Wagner, 2016).


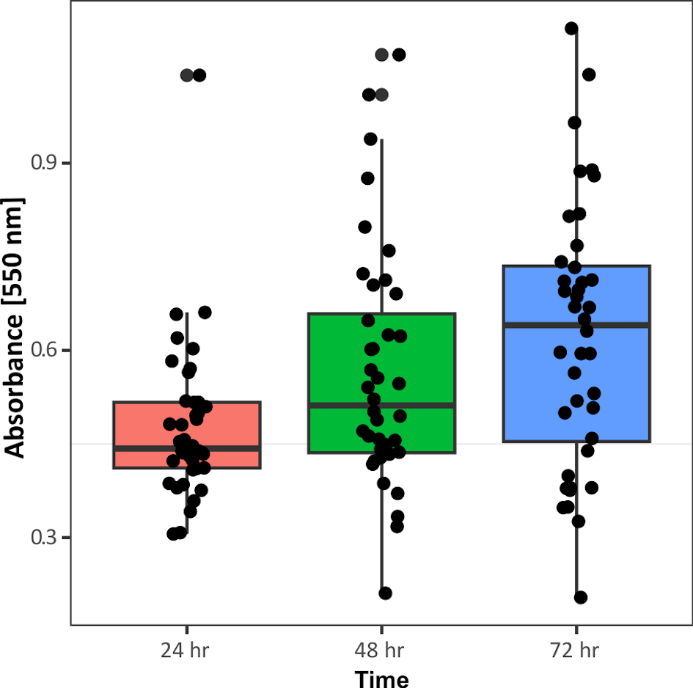


**Supplementary Figure 3**. Biofilm production of *A. hydrophila* INISA09 measured by violet crystal stain according to (O’Toole and Kolter, 1998) at three different times: 24, 48, and 72 hours.

## Supplementary Tables

Supplementary Table 1. *Aeromonas hydrophila* genomes used as references for genomic analyses.

| **RefSeq ID** | **Strain** | **Publication** | **No. Prophages** | **Observations** |
| --- | --- | --- | --- | --- |
| AP019193 | GSH8-2 | (Sekizuka et al., 2019) | 2 |  |
| AP022206 | WP7-S18-ESBL-06 | Unpublished | 2 |  |
| AP022252 | WP8-S18-ESBL-02 | Unpublished | 2 |  |
| AP023398 | KAM330 | Unpublished | 3 |  |
| AP024234 | RIMD111065 | (Yamazaki et al., 2020) | 1 |  |
| CP006579 | 4AK4 | (Gao et al., 2013) | 2 |  |
| CP027804 | KN-Mc-1R2 | (Lim et al., 2020) | 4 |  |
| CP028568 | WCHAH045096 | Unpublished | 3 |  |
| CP038463 | WCX23 | (Liu et al., 2019) | 4 |  |
| CP038465 | 23-C-23 | (Liu et al., 2019) | 4 |  |
| CP043323 | 3206 | Unpublished | 3 |  |
| CP043324 | 2359 | Unpublished | 3 |  |
| CP045220 | Aer_Brac66 | Unpublished | 1 |  |
| CP045501 | Aer_Pi25.1HTAS | Unpublished | 5 |  |
| CP045502 | Aer_Brac14A | Unpublished | 2 |  |
| CP046954 | HX-3 | Unpublished | 3 |  |
| CP050850 | Brac6 | Unpublished | 1 |  |
| CP050851 | OnP3.1 | Unpublished | 1 |  |
| CP050994 | NEB724 | (Anton et al., 2021) | 1 |  |
| CP053859 | B11 | Unpublished | 0 |  |
| CP053883 | 4960 | (Wang et al., 2021) | 3 |  |
| CP053884 | 3924 | (Wang et al., 2021) | 1 |  |
| CP064382 | PartN-Ahydrophila-RM8376 | Unpublished | 3 |  |
| CP065651 | FDAARGOS_916 | Unpublished | 3 |  |
| CP083944 | CSUSB2 | Unpublished | 4 |  |
| CP084353 | 71317 | Unpublished | 0 |  |
| CP084581 | Ah27 | Unpublished | 5 |  |
| CP092708 | S-P-C-021.01 | Unpublished | 2 |  |
| CP092709 | S-P-C-022.01 | Unpublished | 2 |  |
| CP092906 | LP0103 | (Bureros et al., 2022) | 3 |  |
| CP093308 | AC185 | Unpublished | 2 |  |
| CP093309 | AC133 | Unpublished | 4 |  |
| CP094267 | A008N2 | Unpublished | - | Genome without annotation |
| CP102110 | GD21SC2284TT | Unpublished | 3 |  |
| NC_021290 | ML09-119 | (Tekedar et al., 2013) | 3 |  |
| NZ_CP006883 | J-1 | Unpublished | 4 |  |
| NZ_CP007518.2 | YL17 | (Lim et al., 2016) | 0 |  |
| NZ_CP007566 | AL09-71 | (Pridgeon et al., 2014b) | 3 |  |
| NZ_CP007576 | PC-104A | (Pridgeon et al., 2014a) | 3 |  |
| NZ_CP010947 | AL06-06 | (Tekedar et al., 2015) | 3 |  |
| NZ_CP011100 | AH10 | Unpublished | 4 |  |
| NZ_CP013178 | JBN2301 | (Yang et al., 2016) | 6 |  |
| NZ_CP013965 | D4 | Unpublished | 4 |  |
| NZ_CP016380 | AHNIH1 | (Hughes et al., 2016) | 1 |  |
| NZ_CP016392 | GYK1 | Unpublished | 2 |  |
| NZ_CP016989 | ZYAH72 | (Li et al., 2021) | 5 |  |
| NZ_CP018201 | MX16A | Unpublished | 2 |  |
| NZ_CP046954 | HX-3 | Unpublished | 3 |  |
| NZ_CP050012 | LHW39 | Unpublished | 5 |  |
| NZ_CP053885 | 3019 | (Wang et al., 2021) | NA | Error running the software |
| NZ_CP084352 | 71339 | Unpublished | 2 |  |
| NZ_CP095280 | Ah2111 | (Xu et al., 2022) | 3 |  |

Supplementary Table 2. Disk diffusion results for different antimicrobials tested on the strain *Aeromonas hydrophila* INISA09.

| **Therapeutic class** | **Antibiotic** | **Tested concentration (µg)** | **Inhibition halo**  **(mm)** |
| --- | --- | --- | --- |
| Aminoglycoside | Gentamicin | 80 | 22 |
|  | Streptomycin | 500 | 26.5 |
| Cephalosporine | Ceftazidime | 500 | 37.8 |
| Dihydropyrimidine | Trimethoprim | 300 | 34.5 |
| Epoxide | Fosfomycin | 300 | 37.1 |
| Glycopeptide | Vancomycin* | 200 | 16 |
| Lipopeptide | Daptomycin* | 40 | 0 |
| Penicillin | Amoxicillin* | 20 480 | 0 |
|  | Ampicillin* | 1 000 | 0 |
| Phenicol | Chloramphenicol | 300 | 40 |
| Polymyxin | Colistin | 200 | 17.4 |
| Quaternary ammonium | BAC | 120 000 | 8 |
| Quinolone | Ciprofloxacin | 10 | 30 |
| Rifamycin | Rifampicin* | 620 | 27 |
| Tetracycline | Doxycycline | 2000 | 25.5 |
|  | Tetracycline | 150 | 35.5 |

* Resistant phenotype

# References

Anton, B. P., Fomenkov, A., Wu, V., and Roberts, R. J. (2021). Genome-wide identification of 5-methylcytosine sites in bacterial genomes by high-throughput sequencing of MspJI restriction fragments. *PLoS One* 16, e0247541. doi: 10.1371/journal.pone.0247541.

Bureros, K. J., Chiu, Y.-C., Liou, C.-Y., Ma, C.-Y., and Wang, L.-C. (2022). Complete Genome Sequence of a Suckermouth Catfish Outbreak Isolate, Aeromonas hydrophila Strain LP0103. *Microbiol. Resour. Announc.* 11. doi: 10.1128/mra.00408-22.

Gao, X., Jian, J., Li, W.-J., Yang, Y.-C., Shen, X.-W., Sun, Z.-R., et al. (2013). Genomic study of polyhydroxyalkanoates producing Aeromonas hydrophila 4AK4. *Appl. Microbiol. Biotechnol.* 97, 9099–9109. doi: 10.1007/s00253-013-5189-y.

Hughes, H. Y., Conlan, S. P., Lau, A. F., Dekker, J. P., Michelin, A. V., Youn, J.-H., et al. (2016). Detection and Whole-Genome Sequencing of Carbapenemase-Producing Aeromonas hydrophila Isolates from Routine Perirectal Surveillance Culture. *J. Clin. Microbiol.* 54, 1167–1170. doi: 10.1128/JCM.03229-15.

Jain, C., Rodriguez-R, L. M., Phillippy, A. M., Konstantinidis, K. T., and Aluru, S. (2018). High throughput ANI analysis of 90K prokaryotic genomes reveals clear species boundaries. *Nat. Commun.* 9, 5114. doi: 10.1038/s41467-018-07641-9.

Li, J., Ma, S., Li, Z., Yu, W., Zhou, P., Ye, X., et al. (2021). Construction and Characterization of an Aeromonas hydrophila Multi-Gene Deletion Strain and Evaluation of Its Potential as a Live-Attenuated Vaccine in Grass Carp. *Vaccines* 9, 451. doi: 10.3390/vaccines9050451.

Lim, S., Lee, D., Kim, Y., Park, S., Kwon, H., Han, J., et al. (2020). Complete genome sequence of multidrug-resistant *Aeromonas hydrophila* strain KN-Mc-1R2 isolated from the wild nutria (*Myocastor coypus*) in Korea. *Korean J. Microbiol.* 56, 340–342. Available at: http://www.kjom.org/journal/view.html?doi=10.7845/kjm.2020.0078.

Lim, Y.-L., Roberts, R. J., Ee, R., Yin, W.-F., and Chan, K.-G. (2016). Complete Genome Sequence and Methylome Analysis of Aeromonas hydrophila Strain YL17, Isolated from a Compost Pile. *Genome Announc.* 4, 1–2. doi: 10.1128/genomeA.00060-16.

Liu, J., Xie, L., Zhao, D., Yang, T., Hu, Y., Sun, Z., et al. (2019). A fatal diarrhoea outbreak in farm‐raised Deinagkistrodon acutu s in China is newly linked to potentially zoonotic Aeromonas hydrophila. *Transbound. Emerg. Dis.* 66, 287–298. doi: 10.1111/tbed.13020.

O’Toole, G. A., and Kolter, R. (1998). Initiation of biofilm formation in Pseudomonas fluorescens WCS365 proceeds via multiple, convergent signalling pathways: A genetic analysis. *Mol. Microbiol.* 28, 449–461. doi: 10.1046/j.1365-2958.1998.00797.x.

Pridgeon, J. W., Zhang, D., and Zhang, L. (2014a). Complete Genome Sequence of a Moderately Virulent Aeromonas hydrophila Strain, pc104A, Isolated from Soil of a Catfish Pond in West Alabama. *Genome Announc.* 2, 94–95. doi: 10.1128/genomeA.00554-14.

Pridgeon, J. W., Zhang, D., and Zhang, L. (2014b). Complete Genome Sequence of the Highly Virulent Aeromonas hydrophila AL09-71 Isolated from Diseased Channel Catfish in West Alabama. *Genome Announc.* 2, 9–10. doi: 10.1128/genomeA.00450-14.

Sekizuka, T., Inamine, Y., Segawa, T., Hashino, M., Yatsu, K., and Kuroda, M. (2019). Potential KPC‐2 carbapenemase reservoir of environmental Aeromonas hydrophila and Aeromonas caviae isolates from the effluent of an urban wastewater treatment plant in Japan. *Environ. Microbiol. Rep.* 11, 589–597. doi: 10.1111/1758-2229.12772.

Sprouffske, K., and Wagner, A. (2016). Growthcurver: an R package for obtaining interpretable metrics from microbial growth curves. *BMC Bioinformatics* 17, 172. doi: 10.1186/s12859-016-1016-7.

Tekedar, H. C., Karsi, A., Akgul, A., Kalindamar, S., Waldbieser, G. C., Sonstegard, T., et al. (2015). Complete Genome Sequence of Fish Pathogen Aeromonas hydrophila AL06-06. *Genome Announc.* 3, 9–10. doi: 10.1128/genomeA.00368-15.

Tekedar, H. C., Waldbieser, G. C., Karsi, A., Liles, M. R., Griffin, M. J., Vamenta, S., et al. (2013). Complete Genome Sequence of a Channel Catfish Epidemic Isolate, Aeromonas hydrophila Strain ML09-119. *Genome Announc.* 1, 1–2. doi: 10.1128/genomeA.00755-13.

Wang, Y., Hou, N., Rasooly, R., Gu, Y., and He, X. (2021). Prevalence and Genetic Analysis of Chromosomal mcr-3/7 in Aeromonas From U.S. Animal-Derived Samples. *Front. Microbiol.* 12, 1–10. doi: 10.3389/fmicb.2021.667406.

Xu, Z., Shen, W., Zhang, R., and Cai, J. (2022). Clonal Dissemination of Aeromonas hydrophila With Binary Carriage of blaKPC-2-Bearing Plasmids in a Chinese Hospital. *Front. Microbiol.* 13. doi: 10.3389/fmicb.2022.918561.

Yamazaki, K., Kashimoto, T., Niwano, A., Yamasaki, M., Nomura, M., Akeda, Y., et al. (2020). Expansion of Necrosis Depending on Hybrid Motor-Driven Motility of Aeromonas hydrophila in a Murine Wound Infection Model. *Microorganisms* 9, 10. doi: 10.3390/microorganisms9010010.

Yang, W., Li, N., Li, M., Zhang, D., and An, G. (2016). Complete Genome Sequence of Fish Pathogen Aeromonas hydrophila JBN2301. *Genome Announc.* 4, 16–17. doi: 10.1128/genomeA.01615-15.
